# *Fallopia japonica*, a Natural Modulator, Can Overcome Multidrug Resistance in Cancer Cells

**DOI:** 10.1155/2015/868424

**Published:** 2015-08-06

**Authors:** Safaa Yehia Eid, Mahmoud Zaki El-Readi, Mohamed Lotfy Ashour, Michael Wink

**Affiliations:** ^1^Department of Biochemistry, Faculty of Medicine, Umm Al-Qura University, Makkah, Saudi Arabia; ^2^Institute of Pharmacy and Molecular Biotechnology, Heidelberg University, Im Neuenheimer Feld 364, 69120 Heidelberg, Germany; ^3^Department of Biochemistry, Faculty of Pharmacy, Al-Azhar University, Assiut 71524, Egypt; ^4^Department of Pharmacognosy, Faculty of Pharmacy, Ain Shams University, Cairo, Egypt

## Abstract

Resistance of cancer cells to chemotherapy is controlled by the decrease of intracellular drug accumulation, increase of detoxification, and diminished propensity of cancer cells to undergo apoptosis. ATP-binding cassette (ABC) membrane transporters with intracellular metabolic enzymes contribute to the complex and unresolved phenomenon of multidrug resistance (MDR). Natural products as alternative medicine have great potential to discover new MDR inhibitors with diverse modes of action. In this study, we characterized several extracts of traditional Chinese medicine (TCM) plants (*N* = 16) for their interaction with ABC transporters, cytochrome P3A4 (CYP3A4), and glutathione-S-transferase (GST) activities and their cytotoxic effect on different cancer cell lines. *Fallopia japonica* (FJ) (Polygonaceae) shows potent inhibitory effect on CYP3A4 P-glycoprotein activity about 1.8-fold when compared to verapamil as positive control. FJ shows significant inhibitory effect (39.81%) compared with the known inhibitor ketoconazole and 100 *μ*g/mL inhibited GST activity to 14 *μ*mol/min/mL. FJ shows moderate cytotoxicity in human Caco-2, HepG-2, and HeLa cell lines; IC_50_ values were 630.98, 198.80, and 317.37 *µ*g/mL, respectively. LC-ESI-MS were used to identify and quantify the most abundant compounds, emodin, polydatin, and resveratrol, in the most active extract of FJ. Here, we present the prospect of using *Fallopia japonica* as natural products to modulate the function of ABC drug transporters. We are conducting future study to evaluate the ability of the major active secondary metabolites of *Fallopia japonica* to modulate MDR and their impact in case of failure of chemotherapy.

## 1. Introduction

Increased cancer mortality and the high cost of treatment spur a continued search for better anticancer drugs. In recent decades, natural compounds have attracted considerable attention as cancer chemopreventive agents and as cancer therapeutics [1]. Some of the most effective cancer treatments to date are natural products or compounds derived from natural products [2]. The first natural product used as an anticancer compound was podophyllotoxin isolated from* Podophyllum peltatum* in 1947. Later, etoposide and teniposide (Chemical derivatives), vinca alkaloids (vinblastine and vincristine), and paclitaxel (Taxol) were discovered as active principle of* Taxus brevifolia* [3]. Natural products and their synthetic derivatives comprise over 77% (63/81) of the approved anticancer drug candidates developed between 1981 and 2006 [4]. This combined percentage highlights the importance of natural products to drug development.

Traditional Chinese medicine (TCM) has a long history of using plant combinations in alleviating and curing the symptoms of many diseases. People are used to get their medication directly from herbal stores or local healers in the form of multicomponents mixtures either to augment the activity of each other or to reduce some of the side effects. TCM had an important role in the 11th Five-Year Plan on National Development of Economy and Society (2006–2010) established by the Chinese government. Food and Drug Administration (FDA) USA has approved the uses of the herbal mixtures; therefore, the sales rates of TCM products were elevated [5]. In addition, all over the world, many pharmaceutical companies have increased interest in herbal medicine after this approval [6]. On the other hand, the new mechanism-based approach informs us that many different events contribute to the eventual success of cancer [7]. Any single drug can at best target a small number of these events, leaving the rest to occur uninterrupted [8]. Moreover, we know that cancer cells have some ability to adapt or be resistant to therapy. We can imagine that a cancer cell can adapt better to one or a few interrupted events than to many.

To overcome this problem, it is necessary to use multiple compounds in combination. TCM are ideally suited for this type of application; TCM drugs are active at reasonable concentrations, and yet their mild nature allows a variety of large combinations to be used safely [9].

Cancer cells can develop resistance not only to one drug but also to entire classes of drugs with similar mechanisms of action. After such resistance is established, some cells even become cross-resistant to drugs, which are structurally and mechanistically unrelated; this phenomenon is known as multidrug resistance (MDR). Multidrug resistance (MDR) against anticancer drugs is a major problem in chemotherapy with 30–80% of cancer patients developing resistance to chemotherapeutical drugs [10]. Thus, counteracting drug resistance is crucial to provide the best treatment. Mechanisms of drug resistance involving ATP-dependent efflux pumps, belonging to ABC transporters (P-gp/MDR1, MRP, and BCRP), although the most thoroughly characterized, are not the only means by which drug resistance can arise within tumor cells. Clinical studies investigating other drug-resistance mechanisms (called nonclassical MDR) are fewer in number but are not less important. These nontransport mechanisms affect multiple drug classes. This type of resistance can be caused by the altered activity of specific enzyme systems such as CYP3A4 and GST, which can decrease the cytotoxic activity of drugs in a manner independent of intracellular drug concentrations [11–13]. The various causes of drug resistance can work simultaneously, increasing the resistance in a multifactorial manner [14]. For example, the simultaneous induction of CYP3A4, GST, and MDR1 was observed [15, 16]. This type of multidrug resistance can be induced after exposure to any drug. Recent evidence indicates that certain nuclear receptors, such as pregnane X receptor (PXR), might be involved in mediating this response to environmental stress while also acting in regulating metabolic enzymes (e.g., CYP3A4 and GST) and ABC transporters (e.g., MDR1 and MRP) [17, 18].

In living system, xenobiotic metabolism involved three main phases:* phase I* which is responsible for the transformation of the substrates into polar metabolites through a hydroxylation reaction,* phase 2* which is related mainly to conjugation with highly soluble glucuronides to facilitate their excretion, and finally phase 3 which are associated with the cleaning pump P-glycoprotein that is accountable for pumping of the metabolized substrates out of the cells.

The cytochrome P450 (CYP) enzymes are the primary (phase I) enzyme system involved in the oxidative metabolism of a wide variety of xenobiotics in the body and their elimination. This metabolizing system has unsurprisingly been associated with a majority of the metabolism-related drug-drug interactions known to date [19]. Drug metabolism in the human liver involved more than fifteen different CYP enzyme isoforms; however, of these, CYP3A4 is considered the most important isoform. It metabolizes more than 50% of the drugs in the liver [20].

Usually lipophilic substances undergo further conjugation in phase II with glucuronic acid, or glutathione. GST is the most famous enzyme involved in this phase, which catalyzes the conjugation of reduced glutathione to electrophilic centers on a wide variety of substrates. These biotransformations enhance the dissolution of the substrates in the cellular sap and hence increase their elimination out of the cells [21].

In this context, and based on the fact that most of the TCM drugs are sold as over-the-counter (OTC) drugs and rare reports could be found with regards to their possible interactions with the metabolizing enzymes, it was very important to investigate the potential interference of 17 commonly used TCM plants on P-gp as well as CYP3A4 and GST enzymes. Besides, their cytotoxicity on different cell lines including the colorectal carcinoma (Caco-2), hepatocellular carcinoma (HepG-2), and cervical carcinoma (HeLa) cells was also evaluated. Characterization and profiling of the most active extract of* Fallopia japonica* (Polygonaceae) were carried out in order to correlate the activity with their secondary metabolites contents.

## 2. Materials and Methods

### 2.1. Plant Material Identification

Plant samples were obtained commercially and identified using DNA barcoding methods [22]. Briefly, the plant DNA was isolated using the phenol chloroform extraction method. A 700 bp fragment of the ribulose-bisphosphate carboxylase* gene* (*rbc*L) was amplified using PCR. The PCR product was sequenced and the identity of the plant species was confirmed (on either the genus or the species level) by comparing the sequence with database entries of authentic species using BioEdit. The genetic distance of the sequenced species to the species of the databases was determined using MEGA 4. This part of the work was performed by Florian Herrmann, IPMB Heidelberg University.

### 2.2. Preparation of the Plant Methanolic Extracts

Different plant materials (100 g) were extracted 5–10 times with methanol until the colored compounds were completely removed from the plant powders. The extracts obtained were filtered, and the total methanol extracts were dried over anhydrous sodium sulphate and evaporated until becoming dry under a vacuum at 45°C. The dried extract was resolved in 10 mL methanol. Portions of the extracts were dried completely in vacuum and the weight of the remaining dry extracts was determined (e.g., in FJ 12.5 g/100 g dry weight). Dried extracts were dissolved in DMSO for the experiments. The experiments were carried out with freshly prepared extracts.

### 2.3. Cell Lines

Caco-2 cells (DSMZ No. ACC 169), HepG-2 (DSMZ No. ACC 180), and HeLa cells (DSMZ No. ACC 57) were maintained in DMEM complete medium (L-glutamine, 10% heat-inactivated fetal bovine serum (FBS), 100 U/mL penicillin, and 100 *μ*g/mL streptomycin) and in addition, 1 mM sodium pyruvate and 1% nonessential amino acids were added to Caco-2 medium. Cells were grown at 37°C in a humidified atmosphere of 5% CO_2_. All experiments were performed with cells in the logarithmic growth phase. The Caco-2 cells described were an ideal model for studying MDR because they highly express ABC transporter proteins, including MDR1 (P-gp), MRP1, and BCRP.

### 2.4. Cytotoxicity Assay

The MTT cytotoxicity assay is widely used, particularly in the field of drug development [23]. Briefly, cells were seeded in 96-well plates with a density of 2 × 10^4^ cells/well. The cells were treated with various concentrations of TCM extracts (up to 4 mg/mL) for 24 h. Then, 0.5 mg/mL MTT was added to each well and incubated for 4 h. The formed formazan crystals were dissolved in DMSO. Absorbance was detected at 570 nm using Tecan Safire II (Crailsheim, Germany).

### 2.5. ABC Transporter Activity

ABC transporter activities of the TCM extracts were determined using rhodamine 123 (Rho123). Rho123 is a known substrate, not only for P-gp but also for MRP1 [24]. Rho123 is readily effluxed in MDR-overexpressing cancer cells. Caco-2 cells: cells were seeded at 2 × 10^3^ cells/well in 96-well plates and cultured under standard conditions until a confluent monolayer was formed (by day 6) as determined by light microscopy. After washing, cells were preincubated for 30 min at 37°C with different concentrations of test samples in order to determine dose dependence. Rho123 (1 *μ*g/mL) was then added and the cells were further incubated for 90 min at 37°C. After washing, the fluorescence of Rho123 was measured at excitation/emission wavelengths of 500/535 nm using a spectrofluorometer Tecan Safire II (Crailsheim, Germany). The fluorescence of test samples themselves was excluded from the calculation of fluorescence intensity.

To quantify and compare the results, the fluorescence intensity of treated cells was normalized by calculating the relative fluorescence intensity (inhibitory efficiency) as the percentage of the positive (verapamil) and untreated control. Inhibitory efficiency was calculated as follows:

(1) Inhibitory efficiency=RFUextract−RFUuntreated controlRFUverapamil−RFUuntreated control%.

RFU_extract_ = fluorescence in the presence of test extract, RFU_verapamil_ = fluorescence in the presence of verapamil, and RFU_untreated  control_ = fluorescence in the absence of the drug. Only values higher than 10% were considered significant.

### 2.6. CYP3A4 Activity Assay

CYP450-Glo (Promega, Mannheim, Germany) was used to detect the effects of the TCM extract on recombinant human CYP3A4 according to the manufacturer instructions [25]. Equal volumes (12.5 *μ*L) of different sample solutions and the reaction mixture containing the CYP3A4 specific substrate (200 *μ*M luciferin 6′ benzyl ether in phosphate buffer pH 7.4) and CYP3A4 (1 pmol/*μ*L) were incubated at room temperature for 10 min. The addition of 25 *μ*L of the NADPH regeneration system (NADP^+^, glucose-6-phosphate, and glucose-6-phosphate dehydrogenase in citrate buffer pH 5.5) initiated the enzyme reaction. After 30 min, 50 *μ*L luciferin was added; 20 min later, the luminescence was recorded using a Tecan Safire II reader. The effects of different extract were evaluated in triplicate relative to blank controls containing 1% DMSO and ketoconazole (10 *μ*M) which was used as a positive control.

### 2.7. Glutathione-S-Transferase Assay

The principle of the GST activity assay is based upon the GST-catalysed reaction between GSH and GST substrate, 1-chloro-2,4-dinitrobenzene (CDNB) as described by Habig et al. [26]. Briefly, untreated and treated HepG-2 cell lysates were used for this assay by preparing a sample with a total 100 *μ*L volume with a standard assay mixture containing 1 mM CDNB, 1 mM reduced glutathione (GSH), and 100 mM PBS (pH 6.5). The reaction was monitored by spectrophotometry at 340 nm. After calculating the GST activity unit (*μ*mol/min/mL) according to manufacturer instructions, the IC_50_ was calculated as % related to the control activity.

### 2.8. Characterization of the* F. japonica* by LC-MS

The MeOH extract of *FJ* (20 mg/mL) was separated by reversed-phase HPLC by injecting 5 *μ*L Rheodyne system. Separation was achieved using a RP-C18e LichroCART 250-4, 5 *μ*m column (Merck, Darmstadt, Germany). The mobile phase consisted of HPLC grade water with 0.5% formic acid (A) and acetonitrile (B). A Merck-Hitachi L-6200A system (Merck, Darmstadt, Germany) was used with a gradient program at a flow rate of 1 mL/min as follows: from 0 to 75% B in 45 minutes and then to 100% in 5 minutes. Mass spectrometry conditions are as follows: a Quattro II system from VG with an ESI interface was used in positive ion and negative ion mode under the following condition: drying and nebulizing gas was nitrogen (N_2_). Capillary temperature was 120°C; capillary voltage was 3.50 kV. Lens voltage was 0.5 kV and cone voltage was 30 V. Full scan mode was in the range of* m/z* 200–800 for which the instrument was set to the following tune parameters: nebulising and drying gas pressure was 350 L/h and 3.5  L/h, respectively. Data were processed using MassLynx 4.0 software (Waters).

## 3. Results

### 3.1. Cytotoxicity of TCM Drugs

The cytotoxicity of TCM drugs (IC_50_) in human Caco-2, HepG-2, and HeLa cell lines is shown in Table 1. The cytotoxic effect of TCM plants varies between cells and species.* Paris polyphylla* (Melanthiaceae) demonstrated the strongest inhibition of proliferation of these cell lines. The IC_50_ values were 53.87, 48.31, and 35.04 *μ*g/mL, respectively, whereas* Cynanchum paniculatum* (Apocynaceae) showed the lowest cytotoxicity for all tested cell lines. IC_50_ values were 2590.48, 2167.54, and 500.50 *μ*g/mL, respectively.* FJ* shows moderate cytotoxicity; IC_50_ values were 630.98, 198.80, and 317.37 *μ*g/mL, respectively. Caco-2 cells were the most resistant cells against all test extracts probability due to their high expression of MDR proteins.

**Figure 1:**
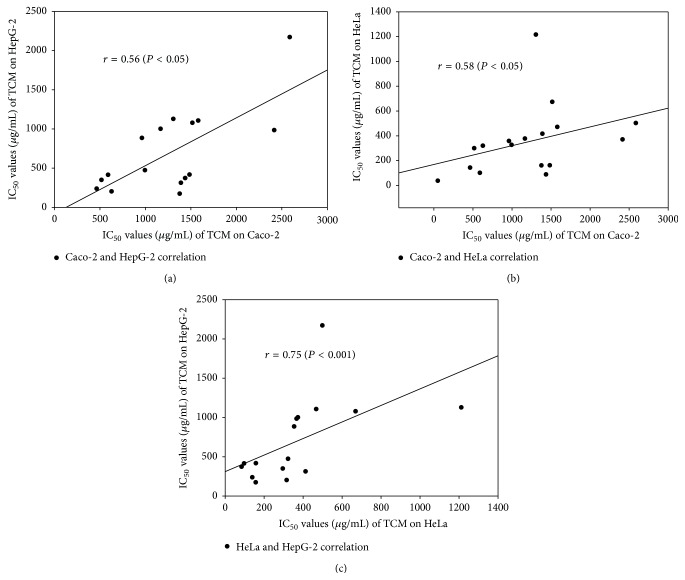
Correlation of cytotoxic effects (IC_50_ values) of TCM plants between different cell lines; cytotoxicity in Caco-2 correlated with HepG-2 (*P* < 0.05) (a) and with HeLa (*P* < 0.01) (b) and HeLa highly significantly correlated with HepG-2 (*P* < 0.001) (c).

A significant correlation between the cytotoxicity of TCM extracts exists between HeLa and HepG-2 with *r* = 0.75 (*P* < 0.001) and between HeLa and Caco-2 cell lines; *r* = 0.58 (*P* < 0.01) (Spearman rank order correlation coefficient). Cytotoxicity of TCM extract was correlated between Caco-2 and HepG-2; *r* = 0.56 (*P* < 0.05). These statistical data refer to the degree of resistance in Caco-2 cell and sensitivity of HeLa and HepG-2, which appear highly correlated to each other in cytotoxic response to test TCM extracts (Figures 1(a)–1(c)).

### 3.2. Effects of TCM Drugs on the Activities of ABC Transporters, CYP3A4, and GST

We first tested the effects of 17 TCM extracts from different families on the accumulation of rhodamine 123 in Caco-2 cells as the model for ABC transporters. As shown in Table 2,* Cymbopogon distans* (Poaceae) and* Ophioglossum vulgatum* (Ophioglossaceae) extracts at 100 *μ*g/mL have no effect on ABC transporter function.

FJ and* Desmodium styracifolium* (Fabaceae) increased the cellular accumulation of the fluorescent substrate about 1.78-fold and 1.71-fold, respectively, when compared to verapamil as positive control.* Sanguisorba officinalis* (Rosaceae) and* Areca catechu* (Arecaceae) show the maximum inhibitory effects on the CYP3A4 enzyme at 100 *μ*g/mL, with 59.96%, and 56.94%, respectively. FJ shows significant inhibitory effect (39.81%) compared with the known inhibitor ketoconazole, which completely inhibits the enzyme activity (Table 2).

The GST specific activities of TCM plants towards 1-chloro-2,4-dinitrobenzene substrate were measured in HepG-2 cells. The majority of the test extracts showed significant GST inhibitory activity at the tested concentration 100 *μ*g/mL. Some extracts have low level GST activity like* Saposhnikovia divaricata* and* Cymbopogon distans*  (~7 and 9 *μ*mol/min/mL), respectively (Table 2). FJ whereas FJ shows a medium GST activity.

Our study target was to search for natural MDR inhibitors. Thus, we selected the most active extract, FJ, for future study to investigate its effect on MDR as a multifactoral phenomenon in details.

### 3.3. LC-ESI-MS of* Fallopia japonica* Plant Extract

**Figure 2:**
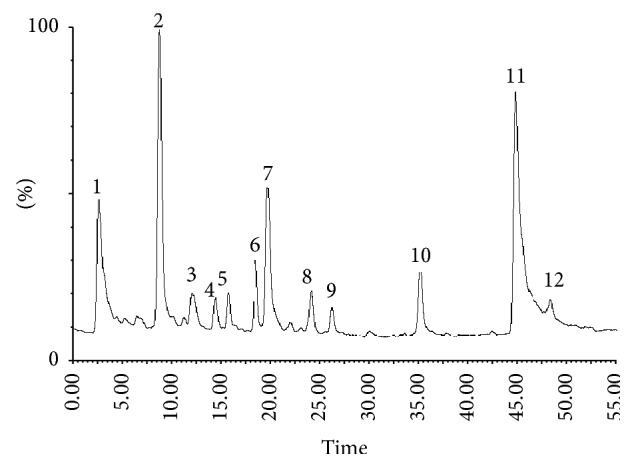
HPLC profile of* F*.* japonica* methanolic extract.

Liquid chromatography (LC) combined with electrospray ionization mass spectrometry (ESI/MS), a relatively new technique rapidly growing in popularity, has been successfully applied to elucidate the structures of the active compounds in herbal extracts [27]. We believe that this technique could be successfully applied to identify the peaks in the HPLC profile of* F. japonica* extract. In this study, by using LC-ESI-MS methods, we identified the major constituents (including water-soluble and lipid-soluble compounds) in the HPLC chromatogram. HPLC profile of authenticated and identified herbal material of FJ was obtained according to the developed HPLC method described above (Figure 2).

The experiments showed that the negative ion mode is more sensitive than the positive ion mode for identifying water-soluble phenolic compounds. The reference standards emodin, polydatin, resveratrol, and rhein were analyzed by direct injection in order to optimize the electrospray ionization ESI-MS conditions.

Eleven characteristic peaks presented in profile were tentatively identified by detailed studies of their ESI-MS spectral data (Table 3) by comparison with published data [28]. The identification of peaks 2, 5, 10, and 11 was further confirmed by comparing their retention time values with those of the standards.

Emodin, polydatin, and resveratrol can be identified unambiguously by comparing the MS data (*m/z* 269, 389, and 227) and the retention times (44.8, 8.8, and 15.7 min), respectively, with those of the literature and authentic compounds [28]. These compounds amount to 29.6, 22.4, and 2.55% of total extract content, respectively (Table 3).

## 4. Discussion

Multidrug resistance to chemotherapeutic drugs is a major problem in tumor treatment. Synergistic interaction between the MDR mechanisms, ABC transporters, metabolic enzymes (CYP3A4 and GST), and apoptosis was observed [29].

The general screening assays reveal the FJ extract to be the most potent inhibitor of ABC transporters, significantly inhibiting metabolic enzymes and cell growth (Tables 1 and 2). Based on the phytochemical profiling, the main components of the methanol extract are resveratrol and its glycoside polydatin, emodin, and its glucoside. These compounds are known inhibitors of both P-gp proteins and are able to reverse multidrug resistance in many cancer cells [30]. Besides, resveratrol is a known inhibitor of different cytochrome isoforms especially CYP1A1, CYP1B1, and CYP3A4 [31, 32].

Emodin and also its glucoside form are known inhibitors of CYP1A1 and CYP1A2 [33]. However, nothing could be found regarding the inhibition of CYP3A4 although other closely related compounds such as rhein can inhibit this metabolizing enzyme.

Among the most active extracts, the Radix Saposhnikoviae (*Saposhnikovia divaricata* Apiaceae) which is widely used as antiphlogistic, analgesic, and antipyretic drug in both Kampo and TCM medicine showed high activity. This activity is based mainly on the presence of highly reactive polyacetylenes, especially panaxynol [34], in addition to the high yield of furanocoumarins and chromones, as divaricatol [35]. Presence of these highly reactive polyacetylenes with their reactive triple bonds can covalently bind to amino and sulphydryl groups of proteins. This alkylation may lead to a conformational change and thus loss of activity [8]. This type of interaction can explain the inhibition of many enzymes such as cytochromes and glutathione S-transferase.

Another example of a plant which potentially affects the activity of drug metabolism especially the P-gp is* Desmodium styracifolium* (Fabaceae). The main modulatory activity can be attributed to its flavonoid/isoflavonoid contents (apigenin, luteolin, and genistin), triterpenoid saponins/sterols (stigmasterol-3-O-*β*-D-glucopyranoside, *β*-daucosterol, and *β*-sitosterol), and alkaloids (desmodimine) [36]. These flavonoids possess one or more phenolic hydroxyl groups. The phenolic hydroxyl groups can partly dissociate under physiological conditions resulting in formation of phenolate ions, which could interact with many enzymes unselectively by forming hydrogen bonds with electronegative atoms of the peptide or ionic bonds with positively charged side chains of basic amino acids, respectively. These noncovalent bonds are quite weak but they could cause a change in protein conformation, which then may lead to protein inactivation [8].

*Areca catechu* (Arecaceae) is the least potent inhibitor of the enzymatic activity in both extracts from our tested plants samples. Despite its neurotoxicity and other toxic hazards [37], the plant is still in use especially in Asia and East Africa as astringent and stimulant and to expel worms and nothing could be traced in literature concerning the possible interactions with the metabolizing enzymes. The activity of both extracts could be ascribed to their contents of tannins with their polyphenolic structures especially Arecatannin A_1_–A_3_ which are abundant in both extracts which are able to bind with the enzyme. Moreover, the alkaloidal content mainly arecoline of the methanol extract could contribute to the overall activity although this action has not been confirmed yet to the CYP3A4 but arecoline shows a significant inhibitory activity against other forms of cytochrome especially CYP1A1 [38].

The same explanation could be also given to the* Sanguisorba officinalis* (Rosaceae). This plant is widely used in Europe and China as astringent and antihaemorrhoidal agent and also in treatment of ulcerative colitis and many skin disorders [39, 40]. The extract contains much hydrolysable and condensed tannins, mainly gallic and ellagic acids derivatives, in addition to flavonoids (rutin), proanthocyanidins, and monodesmosidic triterpenoidal saponins. Although these secondary metabolites show a significant inhibition of the CYP3A4 and there is one report about affecting the pharmacokinetics of ciprofloxacin [41], no reports are found concerning the interaction of the plant extract with the cytochromes.

*Chrysanthemum* (Apiaceae) plants showed higher activities of the extract. The main activity is related to its content of the triterpenoidal alcohols, which are presented in higher concentrations extracts. Although these plants are widely used in traditional medicine for treatment of fever, inflammations, and many infections [42], only one report deals with the interaction between another* Chrysanthemum* spp. with many cytochrome isoforms where* C. parthenium* extracts modestly inhibited the activity of CYP1A2, CYP2C8, CYP2C9, CYP2C19, and CYP3A4 [43].

In conclusion, TCM plants modulate MDR through their interaction with P-gp, CYP3A4, and GST. This potent effects of TCM extracts may be due to the synergistic interactions of their SMs which have diverse structure and mode of action.* Fallopia japonica* extract shows potent inhibition of ABC transporters and significant inhibiting metabolic enzymes and cell growth. Further study is needed to evaluate the effects of the individual active compounds of* Fallopia japonica* on MDR in cancer cells.

## Figures and Tables

**Table 1:** Cytotoxicity (IC_50_ values *µ*g/mL) of methanolic extracts from TCM plants in Caco-2, HepG-2, and HeLa cells.

Plant Latin name (family)	IC_50_ values		
	Caco-2	HepG-2	HeLa
*Areca catechu* (Arecaceae)	1393.74 ± 120.34	308.66 ± 39.75	414.29 ± 72.72
*Cassia tora* (Fabaceae)	1520.01 ± 123.43	1074.56 ± 110.70	670.94 ± 40.63
*Chrysanthemum indicum* (Asteraceae)	965.23 ± 90.87	880.86 ± 61.35	355.75 ± 16.81
*Cymbopogon distans* (Poaceae)	592.62 ± 15.56	410.52 ± 40.19	98.85 ± 9.02
*Cynanchum paniculatum* (Apocynaceae)	2590.48 ± 290.02	2167.54 ± 199.84	500.50 ± 57.65
*Desmodium styracifolium *(Fabaceae)	998.54 ± 47.89	469.31 ± 17.03	324.39 ± 34.68
*Kadsura longipedunculata* (Schisandraceae)	1439.96 ± 66.47	368.54 ± 30.09	86.11 ± 3.21
*Mentha haplocalyx* (Lamiaceae)	1170.12 ± 40.11	997.45 ± 56.89	375.06 ± 50.61
*Ophioglossum vulgatum* (Ophioglossaceae)	1584.14 ± 38.08	1102.23 ± 76.39	469.06 ± 54.73
*Paris polyphylla* (Melanthiaceae)	53.87 ± 5.67	48.31 ± 4.43	35.04 ± 6.45
*Patrinia scabiosaefolia* (Valerianaceae)	1488.25 ± 68.12	413.30 ± 93.83	159.41 ± 8.56
*Fallopia japonica* (Polygonaceae)	630.98 ± 40.21	198.80 ± 16.80	317.37 ± 22.27
*Sanguisorba officinalis *(Rosaceae)	1380.69 ± 60.77	169.49 ± 17.82	158.53 ± 13.25
*Saposhnikovia divaricata* (Apiaceae)	2420.03 ± 210.54	980.34 ± 89.76	368.20 ± 19.88
*Taxillus chinensis* (Loranthaceae)	1310.34 ± 31.21	1123.65 ± 52.43	1213.42 ± 21.47
*Viola yezoensis* (Violaceae)	520.23 ± 70.43	345.23 ± 34.23	297.53 ± 19.99

**Table 2:** Effect of TCM drugs (100 *µ*g/mL) on activities of ABC transporters, GST, and CYP3A4.

Plant (family)	Metabolic activity		
	MDR inhibition %	GST activity (*μ*mol/min/mL)	CYP3A4 inhibition %
*Areca catechu* (Arecaceae)	1.27 ± 0.13	23.46 ± 3.34	56.94 ± 4.35
*Cassia tora* (Fabaceae)	8.84 ± 0.97	31.81 ± 3.54	16.38 ± 3.08
*Chrysanthemum indicum* (Asteraceae)	9.83 ± 0.89	22.86 ± 1.54	46.18 ± 4.99
*Cymbopogon distans* (Poaceae)	0.28 ± 0.03	9.54 ± 1.02	2.71 ± 1.53
*Cynanchum paniculatum* (Apocynaceae)	19.29 ± 4.32	15.70 ± 1.72	22.62 ± 3.62
*Desmodium styracifolium *(Fabaceae)	171.24 ± 18.54	10.93 ± 1.32	10.22 ± 3.12
*Kadsura longipedunculata* (Schisandraceae)	2.99 ± 0.35	10.14 ± 1.39	48.30 ± 4.70
*Mentha haplocalyx* (Lamiaceae)	8.96 ± 1.00	12.92 ± 1.81	1.19 ± 0.37
*Ophioglossum vulgatum* (Ophioglossaceae)	1.21 ± 0.15	13.91 ± 1.96	24.91 ± 0.54
*Paris polyphylla* (Melanthiaceae)	15.41 ± 2.01	18.88 ± 2.11	2.92 ± 1.18
*Patrinia scabiosaefolia* (Valerianaceae)	6.32 ± 0.82	27.43 ± 1.32	0.61 ± 0.04
*Fallopia japonica* (Polygonaceae)	179.94 ± 18.34	14.11 ± 1.23	39.81 ± 4.68
*Sanguisorba officinalis *(Rosaceae)	35.82 ± 4.65	19.88 ± 3.43	59.96 ± 3.52
*Saposhnikovia divaricata* (Apiaceae)	134.38 ± 15.9	7.35 ± 0.67	8.21 ± 3.14
*Taxillus chinensis* (Loranthaceae)	43.60 ± 5.76	9.94 ± 1.9	47.41 ± 1.07
*Viola yezoensis* (Violaceae)	65.77 ± 6.89	15.31 ± 2.85	24.97 ± 2.75

MDR inhibition calculated as % compared to verapamil as positive control (100%).

Data are means ± S.D. from three independent experiments.

**Table 3:** Identification of secondary metabolites in methanol extract of *F. japonica* by LC-ESI/MS.

Peak	RT (min)	[M−H]^−^ (*m*/*z*)	Other ions (*m*/*z*)	Compound	%
1	2.6	290	—	Unknown	14.2
2	8.8	389	—	Polydatin	22.4
3	12	541	—	Polydatin gallate	3
4	14.4	431	—	Apigenin-7-glucoside	2
5	15.7	227	—	Resveratrol	2.55
6	18.5	407	245_[M − H-glu]^−^_	Torachrysone-8-O-*β*-glucoside	4.23
7	19.7	431	—	Emodin-8-*β*-D-glucoside	11.1
8	24.2	445	283_[M − H-glu]^−^_	Physcion-8-*β*-D-glucoside	2.9
9	26.3	285	—	Hydroxyemodin	1.6
10	35.2	283	—	Rhein	5.2
11	44.8	269	—	Emodin	29.6
12	48.3	283	—	Physcion	1.2

## References

[1] Nobili S., Lippi D., Witort E. (2009). Natural compounds for cancer treatment and prevention. *Pharmacological Research*.

[2] Dias D. A., Urban S., Roessner U. (2012). A historical overview of natural products in drug discovery. *Metabolites*.

[3] Colegate S. M., Molyneux R. J. (2008). *Bioactive Natural Products: Detection, Isolation, and Structural Determination*.

[4] Newman D. J., Cragg G. M. (2007). Natural products as sources of new drugs over the last 25 years. *Journal of Natural Products*.

[5] Wachtel-Galor S., Benzie I. F. F., Benzie I. F. F., Wachtel-Galor S. (2011). Herbal medicine: an introduction to its history, usage, regulation, current trends, and research needs. *Herbal Medicine: Biomolecular and Clinical Aspects*.

[6] Li J. W.-H., Vederas J. C. (2009). Drug discovery and natural products: end of an era or an endless frontier?. *Science*.

[7] Brower V. (2008). Back to nature: extinction of medicinal plants threatens drug discovery. *Journal of the National Cancer Institute*.

[8] Wink M. (2008). Evolutionary advantage and molecular modes of action of multi-component mixtures used in phytomedicine. *Current Drug Metabolism*.

[9] Boik J. (2001). *Natural Compounds in Cancer Therapy*.

[10] Velingkar V. S., Dandekar V. D. (2010). Modulation of P-glycoprotein mediated multidrug resistance (MDR) in cancer using chemosensitizers. *International Journal of Pharmaceutical Sciences and Research*.

[11] Pluen A., Boucher Y., Ramanujan S. (2001). Role of tumor-host interactions in interstitial diffusion of macromolecules: cranial vs. subcutaneous tumors. *Proceedings of the National Academy of Sciences of the United States of America*.

[12] Jain R. K. (2001). Delivery of molecular and cellular medicine to solid tumors. *Advanced Drug Delivery Reviews*.

[13] Mao Z.-P., Zhao L.-J., Zhou S.-H., Liu M.-Q., Tan W.-F., Yao H.-T. (2015). Expression and significance of glucose transporter-1, P-glycoprotein, multidrug resistance-associated protein and glutathione S-transferase-*π* in laryngeal carcinoma. *Oncology Letters*.

[14] Cort A., Ozben T. (2015). Natural product modulators to overcome multidrug resistance in cancer. *Nutrition and Cancer*.

[15] Schuetz E. G., Beck W. T., Schuetz J. D. (1996). Modulators and substrates of *P*-glycoprotein and cytochrome P4503A coordinately up-regulate these proteins in human colon carcinoma cells. *Molecular Pharmacology*.

[16] Awortwe C., Manda V. K., Avonto C. (2015). *Echinacea purpurea* up-regulates CYP1A2, CYP3A4 and MDR1 gene expression by activation of pregnane X receptor pathway. *Xenobiotica*.

[17] Jiang H., Chen K., He J. (2009). Association of pregnane x receptor with multidrug resistance-related protein 3 and its role in human colon cancer chemoresistance. *Journal of Gastrointestinal Surgery*.

[18] Pondugula S. R., Flannery P. C., Abbott K. L. (2015). Diindolylmethane, a naturally occurring compound, induces CYP3A4 and MDR1 gene expression by activating human PXR. *Toxicology Letters*.

[19] Denisov I. G., Grinkova Y. V., Baylon J. L., Tajkhorshid E., Sligar S. G. (2015). Mechanism of drug–drug interactions mediated by human cytochrome P450 CYP3A4 monomer. *Biochemistry*.

[20] Guengerich F. P. (1999). Cytochrome P-450 3A4: regulation and role in drug metabolism. *Annual Review of Pharmacology and Toxicology*.

[21] Strange R. C., Jones P. W., Fryer A. A. (2000). Glutathione S-transferase: genetics and role in toxicology. *Toxicology Letters*.

[22] Doyle J. J., Doyle J. L. (1990). Isolation of plant DNA from fresh tissue. *Focus*.

[23] Carmichael J., DeGraff W. G., Gazdar A. F., Minna J. D., Mitchell J. B. (1987). Evaluation of a tetrazolium-based semiautomated colorimetric assay: assessment of chemosensitivity testing. *Cancer Research*.

[24] Twentyman P. R., Rhodes T., Rayner S. (1994). A comparison of rhodamine 123 accumulation and efflux in cells with P-glycoprotein-mediated and MRP-associated multidrug resistance phenotypes. *European Journal of Cancer*.

[25] Cali J. J., Ma D., Sobol M. (2006). Luminogenic cytochrome P450 assays. *Expert Opinion on Drug Metabolism and Toxicology*.

[26] Habig W. H., Pabst M. J., Fleischner G., Gatmaitan Z., Arias I. M., Jakoby W. B. (1974). The identity of glutathione S transferase B with ligandin, a major binding protein of liver. *Proceedings of the National Academy of Sciences of the United States of America*.

[27] Cai Z., Lee F. S. C., Wang X. R., Yu W. J. (2002). A capsule review of recent studies on the application of mass spectrometry in the analysis of Chinese medicinal herbs. *Journal of Mass Spectrometry*.

[28] Yi T., Zhang H., Cai Z. (2007). Analysis of rhizoma Polygoni cuspidati by HPLC and HPLC-ESI/MS. *Phytochemical Analysis*.

[29] Harmsen S., Meijerman I., Beijnen J. H., Schellens J. H. M. (2007). The role of nuclear receptors in pharmacokinetic drug-drug interactions in oncology. *Cancer Treatment Reviews*.

[30] Wink M., Ashour M. L., El-Readi M. Z. (2012). Secondary metabolites from plants inhibiting ABC transporters and reversing resistance of cancer cells and microbes to cytotoxic and antimicrobial agents. *Frontiers in Microbiology*.

[31] Chan W. K., Delucchi A. B. (2000). Resveratrol, a red wine constituent, is a mechanism-based inactivator of cytochrome P450 3A4. *Life Sciences*.

[32] Chang T. K. H., Chen J., Lee W. B. K. (2001). Differential inhibition and inactivation of human CYP1 enzymes by trans-resveratrol: evidence for mechanism-based inactivation of CYP1A2. *Journal of Pharmacology and Experimental Therapeutics*.

[33] Bhadauria M., Nirala S. K., Shrivastava S. (2009). Emodin reverses CCl_4_ induced hepatic cytochrome P450 (CYP) enzymatic and ultrastructural changes: the *in vivo* evidence. *Hepatology Research*.

[34] Wang C.-N., Shiao Y.-J., Kuo Y.-H., Chen C.-C., Lin Y.-L. (2000). Inducible nitric oxide synthase inhibitors from *Saposhnikovia divaricata* and *Panax quinquefolium*. *Planta Medica*.

[35] Okuyama E., Hasegawa T., Matsushita T., Fujimoto H., Ishibashi M., Yamazaki M. (2001). Analgesic components of Saposhnikovia root (*Saposhnikovia divaricata*). *Chemical & Pharmaceutical Bulletin*.

[36] Kubo T., Hamada S., Nohara T. (1989). Study on the constituents of *Desmodium styracifolium*. *Chemical and Pharmaceutical Bulletin*.

[37] Wink M., van Wyk B.-E. (2008). *Mind-Altering and Poisonous Plants of the World*.

[38] Chang E. E., Miao Z.-F., Lee W.-J. (2007). Arecoline inhibits the 2,3,7,8-tetrachlorodibenzo-p-dioxin-induced cytochrome P450 1A1 activation in human hepatoma cells. *Journal of Hazardous Materials*.

[39] Van Wyk B.-E., Wink M. (2004). *Medicinal Plants of the World: An Illustrated Scientific Guide to Important Medicinal Plants and Their Uses*.

[40] Wu J.-N. (2005). *An Illustrated Chinese Materia Medica*.

[41] Zhu M., Wong P. Y. K., Li R. C. (1999). Influence of *Sanguisorba officinalis*, a mineral-rich plant drug, on the pharmacokinetics of ciprofloxacin in the rat. *Journal of Antimicrobial Chemotherapy*.

[42] Fundukian L. J. (2009). *The Gale Encyclopedia of Alternative Medicine*.

[43] Unger M., Frank A. (2004). Simultaneous determination of the inhibitory potency of herbal extracts on the activity of six major cytochrome P450 enzymes using liquid chromatography/mass spectrometry and automated online extraction. *Rapid Communications in Mass Spectrometry*.

